# *Pseudomonas aeruginosa *β-lactamase induction requires two permeases, AmpG and AmpP

**DOI:** 10.1186/1471-2180-10-328

**Published:** 2010-12-30

**Authors:** Kok-Fai Kong, Alian Aguila, Lisa Schneper, Kalai Mathee

**Affiliations:** 1 Department of Biological Sciences, College of Arts and Sciences, Florida International University, Miami, FL USA; 2 Department of Molecular Microbiology and Infectious Diseases, Herbert Wertheim College of Medicine, Florida International University, Miami, FL USA

## Abstract

**Background:**

In Enterobacteriaceae, β-lactam antibiotic resistance involves murein recycling intermediates. Murein recycling is a complex process with discrete steps taking place in the periplasm and the cytoplasm. The AmpG permease is critical to this process as it transports N-acetylglucosamine anhydrous N-acetylmuramyl peptides across the inner membrane. In Pseudomonadaceae, this intrinsic mechanism remains to be elucidated. Since the mechanism involves two cellular compartments, the characterization of transporters is crucial to establish the link.

**Results:**

*Pseudomonas aeruginosa *PAO1 has two *ampG *paralogs, *PA4218 *(*ampP*) and *PA4393 *(*ampG*). Topology analysis using β-galactosidase and alkaline phosphatase fusions indicates *ampP *and *ampG *encode proteins which possess 10 and 14 transmembrane helices, respectively, that could potentially transport substrates. Both *ampP *and *ampG *are required for maximum expression of β-lactamase, but complementation and kinetic experiments suggest they act independently to play different roles. Mutation of *ampG *affects resistance to a subset of β-lactam antibiotics. Low-levels of β-lactamase induction occur independently of either *ampP *or *ampG*. Both *ampG *and *ampP *are the second members of two independent two-gene operons. Analysis of the *ampG *and *ampP *operon expression using β-galactosidase transcriptional fusions showed that in PAO1, *ampG *operon expression is β-lactam and *ampR*-independent, while *ampP *operon expression is β-lactam and *ampR*-dependent. β-lactam-dependent expression of the *ampP *operon and independent expression of the *ampG *operon is also dependent upon *ampP*.

**Conclusions:**

In *P. aeruginosa*, β-lactamase induction occurs in at least three ways, induction at low β-lactam concentrations by an as yet uncharacterized pathway, at intermediate concentrations by an *ampP *and *ampG *dependent pathway, and at high concentrations where although both *ampP *and *ampG *play a role, *ampG *may be of greater importance. Both *ampP *and *ampG *are required for maximum induction. Similar to *ampC*, *ampP *expression is inducible in an *ampR*-dependent manner. Importantly, *ampP *expression is autoregulated and *ampP *also regulates expression of *ampG*. Both AmpG and AmpP have topologies consistent with functions in transport. Together, these data suggest that the mechanism of β-lactam resistance of *P. aeruginosa *is distinct from well characterized systems in Enterobacteriaceae and involves a highly complicated interaction between these putative permeases and known Amp proteins.

## Background

*Pseudomonas aeruginosa *is a Gram negative opportunistic pathogen. As a frequent colonizer of catheters and the most frequent fatal causative agent of ventilator-assisted pneumonia, it is one of the most common agents in health-care associated infection [1]. Lung deterioration due to chronic infection by *P. aeruginosa *affects patients with chronic obstructive pulmonary disorder and is a leading cause of morbidity and mortality in cystic fibrosis patients [2]. *P. aeruginosa *infection treatment is often difficult because of the organism's intrinsic and acquired antibiotic resistance. This is due to the presence of multidrug efflux pumps [3], low outer membrane permeability [4], hypermutability [5], biofilm formation [6], and β-lactamase expression [7,8].

*P. aeruginosa *has two chromosomally encoded β-lactamases: the PoxB oxacillinase and the AmpC cephalosporinase [8-10]. Much of what is known about AmpC regulation is from studies in *Escherichia coli*, *Citrobacter freundii *and *Enterobacter cloacae*. These studies have elegantly demonstrated that induction of AmpC, the chromosomal β-lactamase, involves *ampR*, *ampD*, and *ampG*, encoding a LysR type transcriptional factor, an amidase, and a permease, respectively [11].

Expression of *C. freundi *AmpR in *E. coli *revealed that during normal physiological growth, AmpR, in the presence of UDP-MurNAc-peptide, binds to the *ampC *promoter and inhibits expression [12]. In *E. coli*, the addition of β-lactam antibiotics causes an increase in the cytosolic 1,6-anhydro-N-acetylmuramyl-L-Ala-γ-D-Glu-meso-diaminopimelic acid (anhMurNAc-tripeptide) concentration, and a decrease in the cytosolic UDP-N-acetylmuramyl-L-Ala-γ-D-Glu-meso-DAP-D-Ala-D-Ala (UDP-MurNAc-pentapeptide) [12]. It was postulated that AmpR can either activate or repress transcription from the *ampC *promoter and that its activity is dependent upon the nature of the bound effector molecule. *In vitro*, in the presence of UDP-MurNAc-pentapeptide, AmpR represses transcription of *ampC*, whereas in the presence of 1,6-anhMurNAc-tripeptide, AmpR activates *ampC *[12]. Thus, it is postulated that binding of 1,6-anhMurNAc-tripeptide alters the conformation of AmpR from the repressive to the activating mode, facilitating the expression of *ampC *[12]. High-levels of 1,6-anhMurNAc-tripeptide accumulate in the absence of *ampD*. AmpD is an amidase that cleaves 1,6-anhMurNAc-tripeptide [13]. Induction of *E. cloacae ampC *was also shown to be *ampG*-dependent [14]. β-lactamase fusion analysis suggests that *E. coli *AmpG contains 10 transmembrane segments and two large cytoplasmic loops [15]. *E. coli *AmpG was shown to transport N-acetylglucosamine-anhydrous N-acetylmuramic acid (GlcNAc-anhMurNAc) and GlcNAc-anhMurNAc-tri, -tetra, and -pentapeptides [16,17].

Comprehensive and elegant studies using Enterobacteriaceae established the paradigm of the β-lactamase induction mechanism. Orthologs of *ampR*, *ampD*, and *ampG *are found in numerous Gram-negative species [18]. Whether similar mechanisms are employed in all these organisms has not been established. It is possible that the induction mechanism could differ. The β-lactamase induction mechanism of *P. aeruginosa *has not been well-defined; however, it is known that *P. aeruginosa *AmpR regulates expression of *ampC *as in other organisms [8-10]. Similar to other systems, *ampR *is located upstream of the *ampC *gene [10]. Additionally, *P. aeruginosa *AmpR controls transcription of the oxacillinase, *poxB*, and several genes involved in virulence [8-10]. Loss of AmpR in *P. aeruginosa *causes a significant elevation in β-lactamase activity and other virulence factors [10]. *P. aeruginosa *also differs from other previously studied systems in that its genome has two *ampG *orthologs, *PA4218 *and *PA4393 *[19]. The current study reveals that these two genes, *PA4218 *and *PA4393*, are required for β-lactamase induction, hence they have been named *ampP *and *ampG*, respectively. Consistent with their putative roles as permeases, fusion analysis suggests that AmpG and AmpP have 14 and 10 transmembrane helices, respectively. Expression of *ampP *is dependent upon AmpR and is autoregulated. Together, these data suggest the distinctiveness of *P. aeruginosa *β-lactamase induction, as it is the first system that potentially involves two permease paralogs, and contribute to the general understanding of the induction mechanism.

## Results

### Genome Sequence Analysis of the *PA4218 *and *PA4393 *Operons

**Figure 1:**
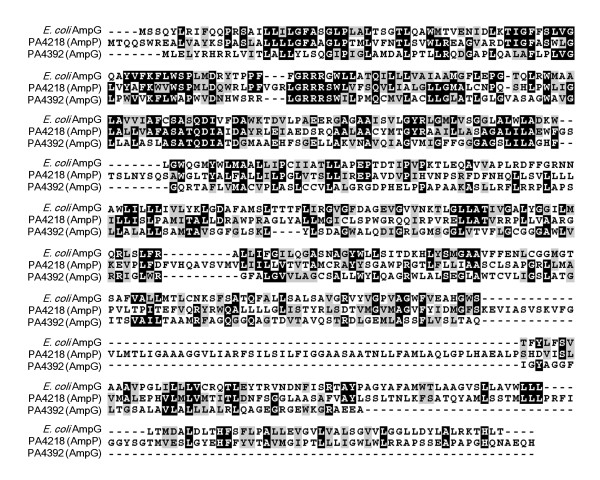
**Alignment of *E. coli *AmpG, PA4218 and PA4393**. The primary sequence of *E. coli *AmpG, PA4218 (AmpP) and PA4393 (AmpG) were used as an input to M-Coffee, which combines multiple sequence alignments using the T-Coffee platform [45,46]. Identical and similar amino acids were shaded black and gray, respectively, using BOXSHADE.

*E. coli *AmpG has been shown to be a permease that transports GlcNAc-anhMurNAc peptides from the periplasm to the cytoplasm [13,17]; however, the AmpG function in *P. aeruginosa *has not been described. BLAST analysis of the *E. coli *AmpG sequence against the six-frame translation of the PAO1 genome identified two open reading frames, *PA4218 *and *PA4393*, with significant homology [20,21]. Global alignment using the Needleman-Wusch algorithm [22] demonstrated that PA4218 is 21.8% identical and 34.8% similar, while PA4393 is 23.2% identical and 34.3% similar to AmpG (Figure 1). The *Pseudomonas *Genome Database identifies *PA4393 *as encoding a putative permease with an alternate name of *ampG*, while *PA4218 *is identified as encoding a probable transporter [23]. Thus, *PA4393 *will be referred to as *ampG *and *PA4218 *as *ampP *(P for permease).

**Figure 2:**
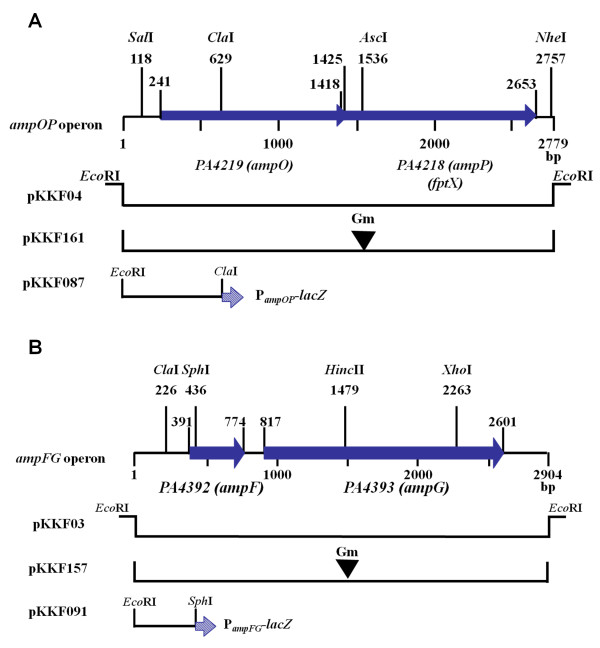
**Physical map of the *ampO-ampP *(A) and *ampF-ampG *(B) loci**. The restriction map is based on PAO1 genome sequence with relevant restriction sites. (A) The 2779-bp *ampO-ampP *fragment has the PAO1 coordinates of 4721496 to 4724275. (B) The 2904-bp *ampF-ampG *fragment corresponds to the PAO1 coordinates of 4921591 to 4924494. The plasmids pKKF03 and pKKF04 are derivatives of pCRII-TOPO (Invitrogen, CA), whereas pKKF157 and pKKF161 are derivatives of pME6030 [41]. The Gm cassette (black inverted triangle) was inserted into the *Hinc*II and *Asc*I sites of pKKF03 and pKKF04, respectively.

Analysis of the sequences around *ampG *and *ampP *revealed that they were in close proximity to two respective upstream ORFs. Based upon sequence analysis, it is likely that *ampG *and *ampP *constitute two two-gene operons with their respective upstream ORFs (Figures 2A and 2B). *PA4219 (ampO) *overlaps the first seven base pairs of *ampP *(Figure 2A). AmpO is a putative seven-transmembrane protein with a strong lipoprotein signal peptide that has a potential cleavage site between amino acids 18 and 19 [23]. The *ampG *gene is located 43 bp downstream from *PA4392 (ampF)*, which encodes a putative protein with a DNA-protein cysteine methyltransferase domain (Figure 2B). The function of this domain remains unknown. No lipoprotein signal was detected in AmpF.

**Figure 3:**
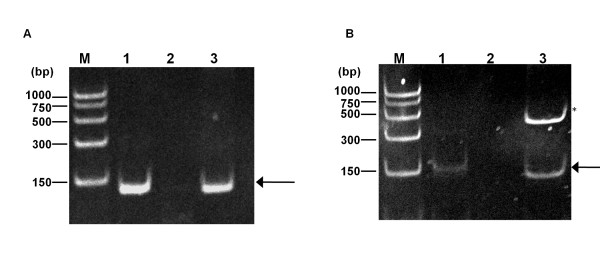
**PCR analysis of *ampFG *and *ampOP *operon cDNA**. Polyacrylamide gel electrophoresis of PCR products of the junctions of the *ampOP *and *ampFG *operons. (A) PCR with primers *PA4392_3*junctionRTF and *PA4392_3*junctionRTR to amplify the *PA4392 *- *PA4393 *intergenic region. (B) PCR with primers *PA4218_9*junctionRTF and *PA4218_9*junctionRTR to amplify the *PA4392 *- *PA4393 *intergenic region. (Panels A and B) Lane M: PCR markers (Promega, Madison, WI). Lane 1, cDNA reaction performed with PAO1 RNA, the appropriate buffer and Superscript RT III. Lane 2, cDNA reaction performed with PAO1 RNA, the appropriate buffer without Superscript RT III. Lane 3, *P. aeruginosa *genomic DNA. The asterisk indicates a nonspecific product. Arrows indicate junction amplicons.

To determine if *ampG *and *ampP *constitute two-gene operons with their upstream ORFs, RNA isolated from PAO1 was analyzed by reverse transcription polymerase chain reaction (PCR) using primers flanking the intergenic (*ampF-ampG*) (Figure 3A) and the overlapping (*ampO-ampP*) region (Figure 3B). The expected amplicon sizes are 136 and 158 bp for the *ampF-G *junction and *ampO-P *junction, respectively [23]. As expected, amplification was observed with genomic DNA (Figures 3A and 3B, Lane 3). In the RNA analyses, PCR products were observed in reverse transcription PCR when the template was prepared in the presence of reverse transcriptase (Figures 3A and 3B, Lane 1), but not in the control reaction when reverse transcriptase was omitted (Figures 3A and 3B, Lane 2). This confirms that *ampO *and *ampP *constitute a two-gene operon and *ampF *and *ampG *constitute another. In addition, reverse transcriptase real time PCR data is in agreement with *ampO *and *ampP *belonging to the same operon and *ampF *and *ampG *comprising another operon (data not shown).

### Topology analysis of AmpG and AmpP

The *ampG *and *ampP *genes encode predicted proteins with 594 and 414 amino acids, isoelectric points of 9.3 and 9.4, and calculated molecular weights of 64.6 kDa and 43.2 kDa, respectively. Hydrophobicity plots predict that AmpG has 16 or 14 predicted transmembrane (TM) helices, depending upon the algorithm used and AmpP has 10 [23]. To determine the membrane topology of AmpG and AmpP, *phoA *or *lacZ *was cloned downstream of the *ampG *and *ampP *genes. The 3'-end of the *ampG *and *ampP *genes were progressively deleted using exonuclease III. At various time-points, the truncated genes were ligated and assayed for PhoA and LacZ activities in *E. coli*. Clones were also sequenced to determine the reporter and *amp *gene junctions.

**Figure 4:**
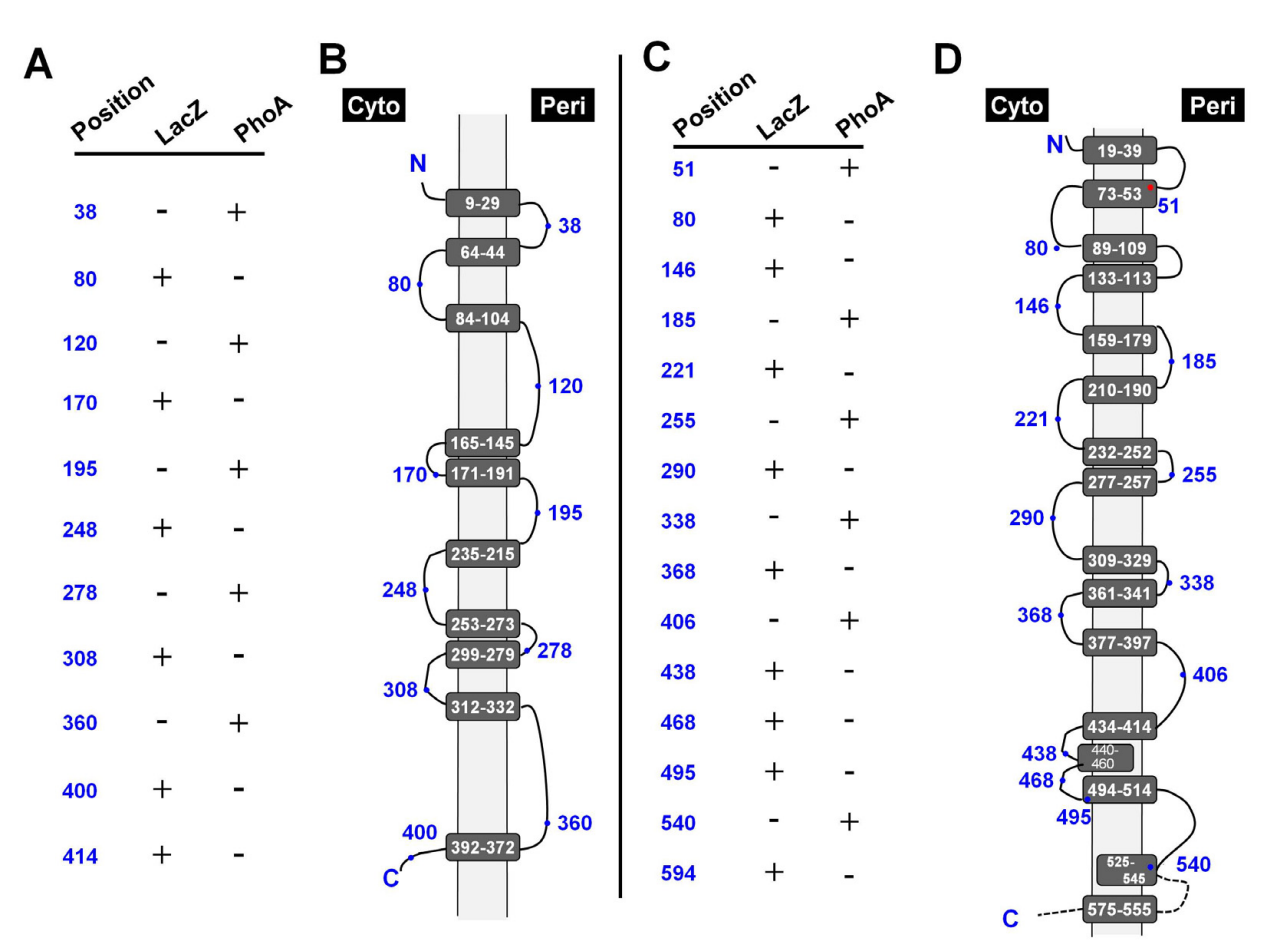
**Topology of AmpP and AmpG**. The topology of AmpP and AmpG was analyzed by in-frame *ampP *and *ampG *fusions to the *lacZ *and *phoA *genes, the cytoplasmic and periplasmic markers, respectively. The corresponding points of fusion and qualitative biochemical results of the β-galactosidase (LacZ) and alkaline phosphatase (PhoA) assays [44] are shown for AmpP (A) and AmpG (C). These results, together with transmembrane domain predictions generated using a Kyte-Doolittle algorithm present in Lasergene 7 (DNASTAR, Madison, WI) were used to predict the topology of AmpP (B) and AmpG (D). Solid lines indicate prediction based upon experimental data, dashed lines indicate regions where more than one possibility exists. Cytoplasm and periplasm are denoted by Cyto and Peri, respectively. Fusion sites are indicated by a dot with the corresponding amino acid number. Putative transmembrane domain boundaries were obtained from Lasergene.

AmpG fusions at amino acids 80, 146, 221, 290, 368, 438, 468, 495, as well as full length were LacZ-positive and PhoA-negative, and fusions at amino acids 51, 185, 255, 338, 406, and 540 were PhoA-positive and LacZ-negative domains, suggesting that AmpG has only 14 TM helices (Figures 4C and 4D). AmpP fusions at amino acids 80, 170, 248, 308, 400 as well as full length were LacZ-positive and PhoA-negative, and fusions at amino acids 38, 120, 195, 278, and 360 were LacZ-negative and PhoA-positive, consistent with 10 TM domains (Figures 4A and 4B).

### β-lactamase activity in strains containing mutations in *ampG *and *ampP*

The failure to induce *C. freundii ampC *in the absence of *E. coli ampG *suggested that AmpG is essential for the induction of chromosomal β-lactamases [24,25]. To ascertain the role of the permeases in *P. aeruginosa*, isogenic *ampG *and *ampP *insertional inactivation mutants were constructed in the prototypic *P. aeruginosa *strain PAO1, referred to as PAO*ampG *and PAO*ampP*, respectively. The β-lactamase activity in the two isogenic mutants, PAO*ampG *and PAO*ampP*, was compared to PAO1. In the absence of β-lactam antibiotics, all strains showed a basal level of β-lactamase activity (Table 1). Upon challenge with 500 μg/ml of benzyl-penicillin, this level was elevated 10-fold (*p *< 0.05) in PAO1 (Table 1). However, the β-lactamase activities of PAO*ampP *and PAO*ampG *remained low in the presence of β-lactam antibiotic, indicating a loss of β-lactamase induction (Table 1). The loss of inducibility in PAO*ampG *could be partially restored by expressing *ampG *in *trans*, whereas the β-lactamase inducibility of PAO*ampP *was completely recovered when *ampP *was supplied in *trans *(Table 1). Both PAO*ampP *and PAO*ampG *mutants had the other copy of the permease gene intact. These observations suggest that *ampG *and *ampP *are individually important members of the β-lactamase induction system. To confirm that *ampG *and *ampP *play independent roles, cross-complementation of PAO*ampP *with pAmpG, and PAO*ampG *with pAmpP was performed. Similar to the mutants, the cross-complemented strains did not show inducible β-lactamase activity (Table 1).

**Table 1:** β-lactamase activity of *P. aeruginosa* PAO1, PAO*ampG* and PAO*ampP* in the absence and presence of β-lactam

Strain and plasmid	Relevant genotypes (supplement in *trans*)	**β-lactamase activity**^**a**^	
		
		Uninduced	Induced^b^
PAO1	*ampG^+^ampP^+^*	22.2 ± 9.7	221.4^c ^± 9.2
PAO*ampG*	*ampG^-^ampP^+^*	20.4 ± 6.2	28.8^d ^± 3.3
PAO*ampP*	*ampG^+^ampP^-^*	4.2 ± 6.2	32.2^d ^± 3.3
PAO*ampG*/pKKF69	*ampG^-^ampP^+ ^*(*ampG^+^)*	8.4 ± 1.4	87.6 ± 14.4
PAO*ampP*/pKKF73	*ampG^+^ampP^- ^*(*ampP^+^)*	8.8 ± 1.8	217.9 ± 35.5
PAO*ampG*/pKKF73	*ampG^-^ampP^+ ^*(*ampP^+^)*	2.1 ± 2.0	14.4 ± 1.9
PAO*ampP*/pKKF69	*ampG^+^ampP^- ^*(*ampG^+^)*	5.3 ± 1.9	10.6 ± 2.7

^a ^Cultures at OD600 of 0.6-0.8 were divided in two. One set was induced with 500 μg/ml benzyl-penicillin for three hours before harvesting. Assays were performed on sonicated lysate using nitrocefin as a chromogenic substrate. One milliunit of β-lactamase is defined as 1 nanomole of nitrocefin hydrolyzed per minute per microgram of protein. Assays were performed in triplicate.

^b ^Induction was carried out using 500 μg/ml benzyl-penicillin

^c ^*p *< 0.05 compared to uninduced PAO1

^d ^*p *< 0.05 compared to induced PAO1

**Figure 5:**
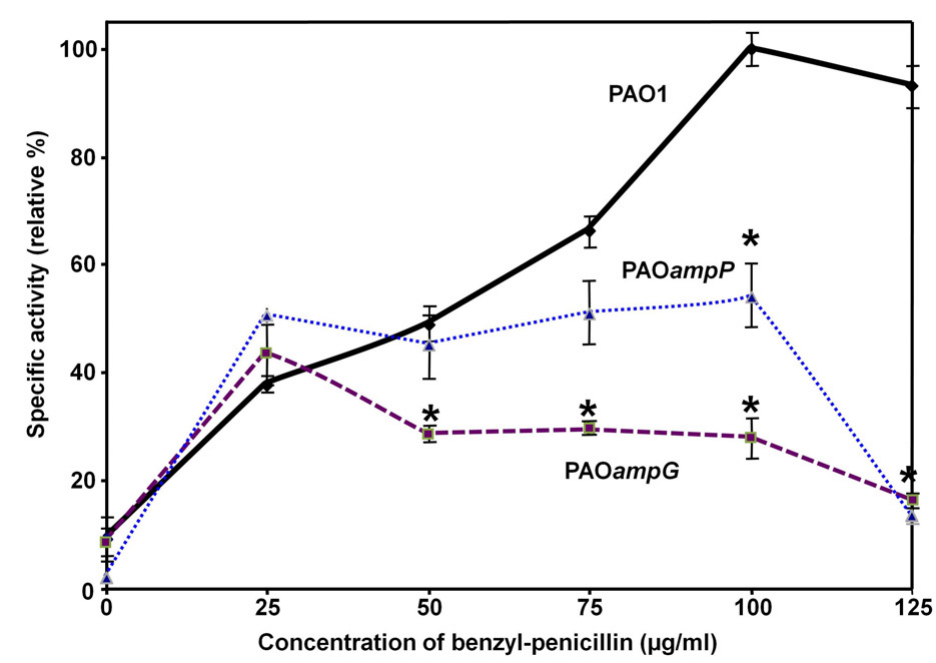
**Relative β-lactamase activity in PAO*ampP *and PAO*ampG *mutants**. Assays were performed on the parental PAO1, and the mutants, PAO*ampP *and PAO*ampG *in the presence of benzyl-penicillin at a concentration gradient of 0 to 125 μg/ml. Cultures at OD_600 _of 0.6-0.8 were induced for three hours before harvesting. Assays were performed on sonicated lysate using nitrocefin as a chromogenic substrate. The β-lactamase activity of PAO1 at 100 μg/ml of benzyl-penicillin was taken as 100%. Each value is the mean of at least three independent experiments. The asterisk refers to *p*-values of < 0.05 with respect to PAO1, which were calculated using the two-tailed Student's *t*-test.

To further understand the role of *ampG *and *ampP *in β-lactamase induction, β-lactamase activity was assayed at different concentrations of benzyl-penicillin in PAO1, PAO*ampG *and PAO*ampP *(Figure 5). Upon encounter with the inducer (25 μg/ml), there was approximately 38% induction (Figure 5). For strain PAO1, this increase in β-lactamase activity continued in a dose-dependent manner until the maximum level of β-lactamase activity was reached when 100 μg/ml of benzyl-penicillin was added (Figure 5). A higher concentration of inducer did not result in a concomitant increase in the expression of the β-lactamase (Figure 5 and data not shown).

In PAO*ampG*, the initial increase of β-lactamase activity was observed at 25 μg/ml, suggesting that this burst of β-lactamase production is *ampG*-independent (Figure 5). However, unlike PAO1, the induction level failed to increase after 25 μg/ml of benzyl-penicillin and even significantly decreased with addition of increased concentrations of benzyl-penicillin (Figure 5).

Mutation of *ampP *also prevented maximum induction of β-lactamase, but the defect was not quite as severe as in PAO*ampG*. In PAO*ampP*, the pattern of β-lactamase induction was very similar to PAO1 at concentrations of benzyl-penicillin up to 50 μg/ml (Figure 5). However, unlike PAO1, addition of benzyl-penicillin at concentrations greater than 50 μg/ml failed to further induce production of β-lactamases (Figure 5). Thus, low induction is independent of *ampG *or *ampP*. The observation that PAO*ampP *exhibited higher levels of β-lactamase expression at higher concentrations of benzyl-penicillin may suggest that *ampG *plays a greater role at higher concentrations of β-lactam.

**Figure 6:**
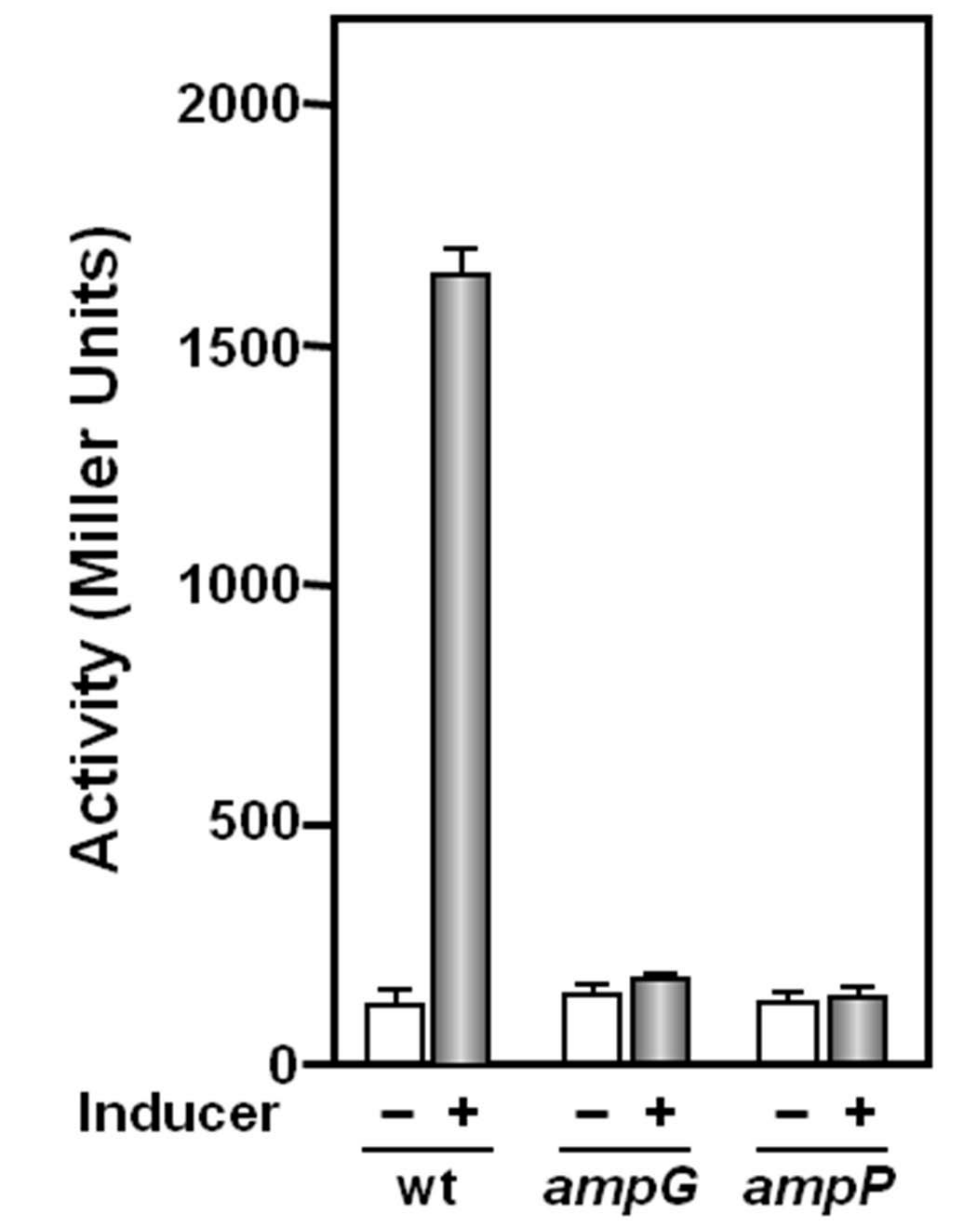
**Activity of the *ampC *promoter**. Promoter activity of the *ampC *gene was analyzed using *lacZ *transcriptional fusions integrated at the *att *locus of PAO1, PAO*ampR*, PAO*ampG *and PAO*ampP *(see Materials and Methods and text for details). Cells were grown to an OD_600 _of 0.6 - 0.8, at which time cultures were divided into two and one set treated with 100 μg/ml benzyl-penicillin. After three hours, cells were harvested and β-galactosidase activity assayed as described [10]. Each value is the mean of at least three independent experiments.

Most of the β-lactamase activity of *P. aeruginosa *can be attributed to AmpC, however, *P. aeruginosa *does contain another chromosomally encoded β-lactamase, PoxB [9,26]. To further analyze if the loss of β-lactamase induction in the PAO*ampG *and PAO*ampP *strains was due to loss of AmpC function, the *ampC *promoter (P_*ampC*_) activity was measured in PAO1, PAO*ampG*, and PAO*ampP*. As expected, upon treatment with benzyl-penicillin, P*_ampC_*-*lacZ *activity increased approximately 15-fold (Figure 6). Benzyl-penicillin dependent induction of P*_ampC_*-*lacZ *was lost in PAO*ampG *or PAO*ampP *(Figure 6).

To further characterize the role of *ampG *and *ampP*, the sensitivity of PAO1, PAO*ampG*, and PAO*ampP *to several β-lactams was determined (Table 2 and data not shown). Inactivation of *ampG *led to a significant decrease in resistance to amoxicillin (> 16-fold) and imipenem (> seven-fold). No difference was observed with ampicillin/sulbactam, cefaclor, cefepime, oxacillin, piperacillin, piperacillin/tazobactam, or ticaricillin/clavulonic acid (data not shown). Inactivation of *ampP *in PAO1 did not alter its resistance profile with these β-lactams (Table 2 and data not shown).

**Table 2:** MICs in PAO1, PAO*ampG* and PAO*ampP* strains

Strain	MIC (μg/ml)	
	Amoxicillin	Imipenem
PAO1	> 256	3
PAO*ampG*	16	0.38
PAO*ampP*	> 256	3

### AmpR regulation of P*_ampFG _*and P*_ampOP_*

**Figure 7:**
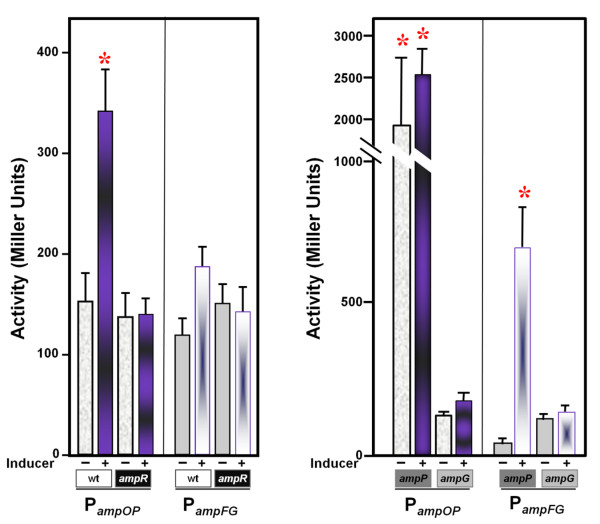
**Activity of the *ampG *and *ampP *promoters**. Promoter activity of the *ampG *and *ampP *genes was analyzed using *lacZ *transcriptional fusions integrated at the *att *locus of PAO1, PAO*ampR*, PAO*ampG *and PAO*ampP *(see Materials and Methods and text for details). Cells were grown to an OD_600 _of 0.6 - 0.8, at which time cultures were divided into two and one set treated with 100 μg/ml benzyl-penicillin. After three hours, cells were harvested and β-galactosidase activity assayed as described [10]. All 16 conditions were assayed at the same time but are divided into two panels for visualization purposes. Each value is the mean of at least three independent experiments. The asterisk refers to *p*-values < 0.05, which were calculated using the two tailed Student's *t*-test.

In inducible *amp *systems, the expression of *ampC *is tightly regulated by the transcription factor, AmpR [27]. In order to investigate the role, if any, of AmpR in the regulation of *P. aeruginosa ampG *and *ampP*, P*_ampFG_-lacZ *and P*_ampOP_-lacZ *promoter fusions were generated and integrated into the chromosome of PAO1 and PAO*ampR *via *attB-attP *site-specific recombination. These constructs are likely to mimic the chromosomal regulation of the *ampFG *and *ampOP *operons. In the absence of inducer in PAO1 and PAO*ampR*, there was a detectable basal level of promoter activity (Figure 7). The expression of the P*_ampOP_-lacZ *promoter fusion was significantly increased in the presence of inducer in the wild-type PAO1, and this induction was lost completely in PAO*ampR *(Figure 7). However, the activity of the P*_ampFG_-lacZ *promoter fusion was comparable to the basal level in the absence and presence of inducer in PAO1 and PAO*ampR*.

### Autoregulation of the *ampG *and *ampP *genes

To determine if *ampG *or *ampP *affected their own or each other's expression, P*_ampFG_-lacZ *and P*_ampOP_-lacZ *promoter fusions were introduced into the chromosomes of PAO*ampP *and PAO*ampG*. Interestingly, the activity of the P*_ampOP_-lacZ *promoter fusion was significantly de-repressed in PAO*ampP *in the absence and presence of inducer (Figure 7). The activity of the P*_ampFG_*-*lacZ *was unchanged in PAO*ampG *in either the absence or presence of benzyl-penicillin. Interestingly, P*_ampFG_*-*lacZ *activity was significantly increased in the presence, but not absence of β-lactam in the PAO*ampP *mutant, suggesting β-lactam-dependent repression of P*_ampG _*by *ampP*. No change of the promoter activity of the *ampOP *operon was observed in the PAO*ampG *mutant.

## Discussion

Members of the Pseudomonadaceae family are intrinsically resistant to β-lactam antibiotics. Earlier reports successfully identified *ampC*, *ampR*, *ampD*, and *ampE *as genes involved in the β-lactamase induction mechanism. However, the question of how chromosomal β-lactamase is induced remains elusive. This study examines the role of two previously uncharacterized *P. aeruginosa *putative permeases.

### *P. aeruginosa *harbors two distinct and independent AmpG orthologues

In Enterobacteriaceae, besides AmpR, AmpD and AmpE, AmpG has also been implicated in the *ampC*-encoded β-lactamase induction, acting as a membrane permease that transports 1,6-anhMurNAc-tripeptide and 1,6-anhMurNAc-pentapeptide [17]. In *P. aeruginosa*, two paralogs, *PA4393/ampG *and *PA4218/ampP*, were found (Figure 1) [28]. Both *ampG *and *ampP *appear to be one member of two independent two-gene operons (Figures 2 and 3). PFAM analysis of AmpP identifies a Major Facilitator Superfamily (MFS1) domain between amino acids 14 and 346, in agreement with a role in transport [23,29,30]. Upstream from *ampP *is *PA4219/ampO*, a gene that has seven putative transmembrane domains [23,31]. Together, these genes form an operon (Figure 3) that is conserved in *P. aeruginosa *PA14, LES, PACS2, and PA2192 [23,32]. In contrast, PFAM analysis of AmpG does not reveal any significant hits, however, there was an insignificant match to the MFS1 domain (E = .00018) [29,30]. The *ampG *gene is downstream from *PA4392/ampF*, which encodes a protein with a putative 6-O-methylguanine-DNA methyltransferase domain [23,33]. These two genes also form an operon (Figure 3) that is conserved in *P. aeruginosa *PA14, LES, and PA7 [23].

The topology of the *E. coli *AmpG permease has been analyzed using β-lactamase fusion proteins [15]. It was shown that AmpG has ten transmembrane domains with the amino- and carboxyl-termini localized to the cytoplasm [15]. In accordance with roles as transporters, AmpG and AmpP have 14 or 16 (depending upon the algorithm used) and 10, respectively predicted TM domains. PhoA and LacZ fusion analysis corroborates the existence of 14 and 10 TM domains in AmpG and AmpP, respectively (Figure 4). In AmpG, the predicted transmembrane helices between amino acids 440 and 460 and either 525 and 545 or 555 to 575 of PA4393 are likely false positives. AmpG fusions at amino acids 438, 468 and 495 indicate that these amino acids are cytoplasmic (Figure 4), suggesting that if the region between amino acids 440 and 460 is membrane associated, it may be an integral monotopic domain. Similarly, AmpG fusions at residues 495 and 594 are cytoplasmic, while that at 540 is periplasmic, suggesting that if the region between amino acids 525 and 545 is membrane associated, it may be an integral monotopic domain. The fusion data indicates that the carboxyl-termini of both AmpG and AmpP are cytoplasmic (Figure 4). Bioinformatic analysis predicts that the amino termini of both proteins are also cytoplasmic. Thus, like *E. coli *AmpG, both the amino and carboxyl termini would be cytoplasmic [15] (Figure 4).

Consistent with a role in transport, AmpP has an MFS domain [23,30]. The Major Facilitator Superfamily domain is present in approximately one-fourth of all known prokaryotic transport proteins [34]. Interestingly, most MFS proteins have 12 TM domains, while AmpP, like *E. coli *AmpG, has only 10 [35]. The topology analysis suggests PAO1 AmpG has 14 TM domains. PAO1 AmpG also has an insignificant MFS1 domain. A few MFS proteins have also been shown to have 14 TM domains [29,35].

### The *ampG *and *ampP *genes are essential for maximum β-lactamase induction

Because of the similarity between AmpG from Enterobacteriaceae and PAO1 AmpG and AmpP, β-lactamase levels of single *ampG *and *ampP *mutant isogenic strains were determined. Although an increase in β-lactamase activity was observed, neither the *ampG *nor *ampP *mutant strain produced the same level of β-lactamase in the presence of benzyl-penicillin as PAO1 (Table 1, Figure 5). Moreover, inactivation of *ampG *or *ampP *abolishes induction of P*_amp_*_*C*_**(Figure 6). This indicates that both *ampG *and *ampP *are essential for chromosomal β-lactamase induction. These genes did not cross-complement or exhibit gene dosage effects indicating that they play different roles in the induction pathway (Table 1). These results are consistent with recent data demonstrating that mutation of *ampG *affects induction of β-lactamase and failure of *ampP *to complement an *ampG *mutation [28]. Furthermore, the analysis using increasing benzyl-penicillin concentrations, shows that *ampP *plays an important role at lower inducer concentrations, whereas *ampG *is crucial at higher concentrations (Figure 5). Mutation of *ampG *affects PAO1 β-lactam resistance (Table 2) [28]. Recent studies by Zhang *et al*., in which deletion of *ampG *results in increased sensitivity to ampicillin [28], are consistent with results presented here (Table 2). In addition, *ampG *inactivation increases imipenem sensitivity (Table 2). Loss of *ampP *(also referred to as *ampGh1*) function did not affect β-lactam sensitivity in either study (Table 2) [28]. AmpP (PA4218) has previously been named FptX due to its homology to RhtX in *Sinorhizobium meliloti *2011 [36]. *PA4219 *does not have a *S. meliloti *orthologue [36]. Mutation of *ampP *in a *P. aeruginosa *CDC5 derivative that produces pyochelin but not pyoverdine, resulted in loss of pyochelin utilization [36]. In agreement with a role in pyochelin utilization, *ampP *is located next to genes involved in pyochelin biosynthesis and transport [23,36]. Thus, the results presented in Table 1 and Figures 5 and 6 demonstrate that *ampP *is involved in β-lactamase induction in addition to its previously characterized role in pyochelin utilization [36].

### Expression of *ampP *is induced by β-lactam addition in the presence of *ampR*

Despite the importance of *ampG *in β-lactamase induction, little is known about its regulation. *E. coli ampG *is also the second gene in a two gene operon. Upstream and divergently transcribed from the *E. coli ampG *operon, is the *bolA *transcriptional regulator [24]. Expression of *bolA *is dependent upon RpoS. Previous studies suggest the expression of the *E. coli ampG *gene is independent of *bolA*, *rpoS *or *ampD *[24]. Neither the *P. aeruginosa ampG *nor *ampP *gene is located near the *bolA *locus [23], thus P*_ampFG _*and P*_ampOP _*-*lacZ *transcriptional fusions were integrated into the chromosome of isogenic PAO1 strains to begin to understand *ampG *and *ampP *regulation.

In light of the requirement of *ampG *and *ampP *for maximum *P. aeruginosa *β-lactamase induction, it was of interest to determine if expression of either was affected by β-lactam addition (Table 1, Figure 5). In the absence of antibiotic, P*_ampFG _*and P*_ampOP _*were constitutively expressed. Expression of P*_ampOP _*significantly increased in the presence of inducer, while P*_ampFG _*did not (Figure 7).

The LysR type transcriptional regulator AmpR induces the expression of the AmpC β-lactamase in the presence of β-lactam antibiotics [27]. AmpR also affects the regulation of additional genes involved in *P. aeruginosa *antibiotic resistance and virulence [10]. Insertional inactivation of *ampR*, did not affect P*_ampFG _*- *lacZ *activity, however, the increase in P*_ampOP_*-*lacZ *activity previously observed upon β-lactam addition was lost in the absence of *ampR *(Figure 7). This indicates that *ampP *expression is regulated by AmpR. Future analyses will determine if this regulation is direct or indirect.

### *ampP *affects regulation of both its own promoter and that of *ampG*

Given that both *ampG *and *ampP *are required for maximum β-lactamase expression, both contain structural elements consistent with roles in transport, and the regulation of *ampP *expression by β-lactam and *ampR*, it was feasible that *ampP *could contribute to its own expression, perhaps by transporting potential effector molecules for AmpR. Indeed, *ampP *does appear to inhibit its own expression, as P*_ampOP _*activity increased ten-fold in PAO*ampP *in the absence, and approximately seven-fold in the presence of β-lactam (Figure 7). Insertional inactivation of *ampP *also resulted in increased expression of P*_ampFG _*in the presence of β-lactam (Figure 7).

### Proposed model for regulation of β-lactamase induction

**Figure 8:**
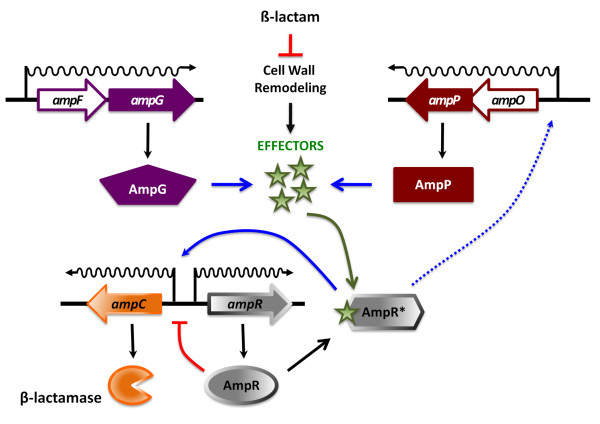
**Model for regulation of AmpC β-lactamase induction by AmpR, AmpP and AmpG in *P. aeruginosa***. In Enterobacteriaceae as well as *P. aerugniosa*, the induction of β-lactamase expression is due to the action of the LysR transcriptional regulator, AmpR. *In vitro *studies suggest that AmpR can act as either a repressor or an activator, depending upon the presence of different peptidoglycan remodelling intermediates. In this study, it is shown that unlike previously characterized systems, *P. aeruginosa *has two putative AmpG permease paralogs, AmpG and AmpP. Expression of AmpP is inducible by β-lactam in an *ampR*-dependent manner. The *ampP *gene also appears to repress its own expression independent of β-lactam through an unknown mechanism. Although not observed to be induced by β-lactam in a PAO1 background, expression of *ampG *also appears to be repressed by *ampP *in the presence of β-lactam (see text for details).

The results presented contribute to what is known concerning β-lactamase induction in *P. aeruginosa*. It is well established that induction of the expression of the AmpC β-lactamase is dependent upon AmpR. Although the exact mechanism has not been well characterized in *P. aeruginosa*, it is believed that the induction is triggered by conversion of AmpR from a repressor to an activator (Figure 8). Evidence from Enterobacteriaceae suggest that this is a result of inhibition of the cell wall remodeling process by β-lactam antibiotics and subsequent accumulation of intermediates which in turn serve as effector molecules for AmpR. Consistent with previous findings suggesting that AmpR acts as a positive regulator of *amp *genes [10], activation of *ampP *expression required the presence of AmpR and β-lactam antibiotic (Figure 7). Based upon glycopeptide accumulation studies in other organisms, these findings suggest that the accumulation of 1,6-anhMurNAc-tripeptide and 1,6-anhMurNAc-pentapeptide in the presence of β-lactam antibiotics activates AmpR that in turn up-regulates the expression of *ampP*. However, *P. aeruginosa *appears to use two non-redundant permeases in β-lactamase induction, suggesting, one may be involved in the import of muramyl peptides and the other in an as yet unknown function. The second permease may be involved in export of muramyl peptides or import of different muramyl peptides. Further studies to determine the identity of these peptides and how they regulate AmpR will be a critical next step in deciphering β-lactam resistance in *P. aeruginosa*.

The *ampP *gene is also auto-regulated via an unknown mechanism. If AmpP performs a similar function as *E. coli *AmpG, the absence of *ampP *would result in the accumulation of the periplasmic pool of GlcNAc-anhMurNAc peptides or the reduction in the cytoplasmic pool of 1,6-anhMurNAc-tripeptide and 1,6-anhMurNAc-pentapeptide alerting the cell that the peptidoglycan recycling process is inhibited. This signalling could result in a positive feedback mechanism that up-regulates the expression of *ampP*. The accumulation of the periplasmic pool of 1,6-anhMurNAc-tripeptide and 1,6-anhMurNAc-pentapeptide in PAO*ampP *is also likely to up-regulate the expression of *P. aeruginosa *PAO1 *ampG *in the presence of β-lactam.

Currently, it is not known if PAO1 AmpG and AmpP function similarly to *E. coli *AmpG, however, like *ampG*, the PAO1 *ampG *and *ampP *are important for β-lactamase induction [14] (Figure 5, Figure 6, Table 1). Moreover, *ampG *and *ampP *are not functionally redundant as both are required for maximum induction. Their expression is also differentially regulated. An *ampP *promoter-*lacZ *fusion exhibited increased activity in the presence of *ampR *and β-lactam or the absence of *ampP*. An *ampG *promoter-*lacZ *fusion was unaffected by the absence or presence of *ampR *or *ampG*. Increased β-galactosidase activity was observed from the *ampG *promoter fusion in the presence of β-lactam in an *ampP *mutant (Figure 7). It is not known if this is dependent upon *ampR*, related to an *ampR*-independent function of *ampP *in β-lactamase induction or the function of *ampP *in pyochelin utilization.

## Conclusions

*P. aeruginosa *appears to have two *ampG *paralogs, *ampG *and *ampP*, which encode proteins with 14 and 10 transmembrane domains. Both are required for maximum induction of chromosomal β-lactamase and induction of the *ampC *promoter. Expression of *ampP *did not restore maximum β-lactamase induced activity in an *ampG *mutation nor did expression of *ampG *complement an *ampP *mutation, indicating that *ampG *and *ampP *have distinct functions in β-lactamase regulation. In addition to being autoregulated, *ampP *is regulated by AmpR and β-lactam. *ampP *is also involved in the regulation of *ampG *in the presence of β-lactam. In summary, the presence of two distinct permeases required for β-lactamase induction suggests that the *P. aeruginosa *β-lactamase resistance mechanism is more complex and distinct from the current paradigm.

## Methods

### Bacterial strains, plasmids and media

Bacterial strains, plasmids and primers employed in this study are shown in Table 3. *E. coli *and *P. aeruginosa *were routinely cultured in Luria-Bertani medium (10 g tryptone, 5 g yeast extract, 5 g NaCl, per liter). *Pseudomonas *Isolation Agar (PIA, Difco) was used in triparental mating experiments. Mueller-Hinton agar (Difco) was used in E-test experiments. Antibiotics, when used, were at the following concentrations (per liter) unless indicated otherwise: ampicillin (Ap) at 50 mg, tetracycline (Tc) at 20 mg, gentamycin (Gm) at 30 mg for *E. coli *and carbenicillin (Cb) at 300 mg, Gm at 300 mg and Tc at 60 mg for *P. aeruginosa*.

**Table 3:** Bacterial strains, plasmids and primers used in this study

Strains/Plasmids	Genotype	Reference
***Escherichia coli***		
TOP10F'	F' {*lacI^q^*, Tn10(Tet^R^)} *mcrA *Δ(*mrr-hsdRMS-mcrBC*) f80d*lacZ*DM15 Δ*lacX74 deoR recA1 araD139 *Δ(*ara-leu*)* 7697 galU galK rpsL *(Str^R^)* endA1 nupG*	Invitrogen
		
***Pseudomonas aeruginosa***		
PAO1	Wild-type	[47]
PKM400	PAO*ampP*::*aacCI; *Gm^R^	This study
PKM500	PAO*ampG*::*aacCI; *Gm^R^	This study
PKM300	PAO*ampR::aacCI; *Gm^R^	[10]
PKM301	PAO*attB*::P*_ampC_*-*lacZ*; Tc^R^	[10]
PKM303	PAO*ampR::aacCI attB*::P*_ampC_*-*lacZ*; Tc^R^Gm^R^	[10]
PKM104	PAO*attB*::P*_ampP_*-*lacZ*; Tc^R^	KKF0290, This study
PKM105	PAO*attB*::P*_ampG_*-*lacZ*; Tc^R^	This study
PKM312	PAO*ampR::aacCI attB*::P*_ampG_*-*lacZ*; Tc^R^Gm^R^	This study
PKM313	PAO*ampR::aacCI attB*::P*_ampP_*-*lacZ*; Tc^R^Gm^R^	This study
PKM404	PAO*ampP::aacCI attB*::P*_ampP_*-*lacZ*; Tc^R^Gm^R^	This study
PKM405	PAO*ampP::aacCI attB*::P*_ampG_*-*lacZ*; Tc^R^Gm^R^	This study
PKM504	PAO*ampG::aacCI attB*::P*_ampP_*-*lacZ*; Tc^R^Gm^R^	This study
PKM505	PAO*ampG::aacCI attB*::P*_ampG_*-*lacZ*; Tc^R^Gm^R^	This study
PKM506	PAO*ampG::aaCI attB*::P_*ampC*_-*lacZ*; Tc^R^Gm^R^	This study
PKM507	PAO*ampP::aaCI attB*::P_*ampC*_-*lacZ*; Tc^R^Gm^R^	This study
		
**Plasmids**		
pCRII-TOPO	Ap^R^, Km^R^; ColE1*ori lacZα*	Invitrogen
pUCGm	Ap^R^, Gm^R^; pUC19 derivative containing gentamycin cassette	[38]
pEX100T	Ap^R^; *sacB oriT*	[39]
pMF54	ColE1-SF broad-host replicon	[48]
pME6030	Tc^R^; *oriV*_pVS1 _*oriV*_p15A _*oriT*	[41]
pRK2013	Km^R^; ColE1*ori*-Tra (RK2)^+^	[40]
pTrcphoA	Ap^R^; low-copy *trc *promoter expression vector carrying the *lacI*^q ^and *phoA*	[43]
pTrclacZ	Ap^R^, low-copy *trc *promoter expression vector carrying the *lacI*^q ^and *lacZ*	[43]
pSJ10	Tc^R^; CTX-*lacZ *fused with *ampC *promoter, P*_ampC_*	[10]
pKKF003	Ap^R^, Km^R^; pCRII-TOPO with a 2904-bp fragment containing PAO1 coordinates 4921591-4924494 (*PA4392-PA4393/ampF-ampG*)	This study
pKKF004	Ap^R^, Km^R^; pCRII-TOPO with a 2779-bp fragment containing PAO1 coordinates *4721496-4724275 *(*PA4217-PA4218/ampO-ampP*)	This study
pKKF069	Tc^R^; pME6030 with a 2904-bp *Eco*RI flanked fragment containing *ampF-ampG*	This study
pKKF073	Tc^R^; pME6030 with a 2779-bp *Eco*RI flanked fragment containing *ampO-ampP*	This study
pKKF087	Tc^R^; CTX-*lacZ *fused with *ampP *promoter, P*_ampP_*	This study
pKKF091	Tc^R^; CTX-*lacZ *fused with *ampG *promoter, P*_ampG_*	This study
pKKF145	Ap^R^, Gm^R^; pCRII-TOPO derivative with *ampP*::*aacCI*	This study
pKKF149	Ap^R^, Gm^R^; pCRII-TOPO derivative with *ampG*::*aacCI*	This study
pKKF157	Ap^R^, Gm^R^; pEX100T derivative with *ampG*::*aacCI*	This study
pKKF161	Ap^R^, Gm^R^; pEX100T derivative with *ampP*::*aacCI*	This study
pAA0115	Ap^R^, pTrcphoA with 1.9 kb *PstI *DNA from pMF54 containing stabilization fragment	This study
pAA0112	Ap^R^, Km^R^; pCRII-TOPO containing a 1797 *ampG *PCR product amplified using primers KKF09 and KKF10	This study
pAA0121	Ap^R^, pAA0115 containing a 1,813 *Eco*RI fragment containing *ampG *from pAA0112	This study
pAA1261	Ap^R^; pAA0121 digested with *Bam*HI and *Sal*I, treated with Klenow and re-ligated to remove an *Xba*I site. Used as basis for Erase-a-base system	This study
pKKF259	Ap^R^; pTrcphoA derivative with a 1797-bp fragment containing *PA4393*	This study
pKKF458	Ap^R^; pTrcphoA derivative with a 1245-bp fragment containing *PA4218*	This study
pKKF459	Ap^R^; pTrclacZ derivative with a 1797-bp fragment containing *PA4393*	This study
pKKF465	Ap^R^; pTrclacZ derivative with a 1245-bp fragment containing *PA4218*	This study
		
**Primers**		
KKF01*ampG*For	5'-TCCAGCTTGACGTCGAGATT-3'	This study
KKF04*ampG*Rev	5'-AGAACATTCTCCTGGCCATGG-3'	This study
KKF05*ampP*For	5'-AACGGCCACGCTAGCAACAC-3'	This study
KKF08*ampP*Rev	5'-GTGGCGCCTGGAGTCTTG-3'	This study
KKF09*ampG*2For	5'-GGGAATTCCATATGACTCAGCAATCCTGG-3'	This study
KKF10*ampG*2Rev	5'-GCTCTAGATGCTCGGCGTTCTGGTGT-3'	This study
KKF13*ampP*2For	5'-TCTAGATCAGGCCTCTTCCGCCCG-3'	This study
KKF14*ampP*2Rev	5'-ATGCTTGAGCTGTACCGCCA-3'	This study
*PA4218_9*junctionRTF	5'-ACCTCACCCTGATCCTCTG-3'	This study
*PA4218_9*junctionRTR	5'-CAGGTAGAGCAACGCCAG-3'	This study
*PA4392_3*junctionRTF	5'-CAACGACAGGGTGGACATAC-3'	This study
*PA4392_3*junctionRTR	5'-GAGACTTGTAGGCGACCAG-3'	This study
(NS)_5_RandomPrimer	5'-NSNSNSNSNS-3'	[49]

### DNA manipulations

Standard procedures in molecular biology were performed as previously described [37].

### Insertional inactivation of the *ampG *and *ampP *genes

A 2904-bp *ampG *fragment was PCR-amplified from PAO1 genomic DNA using KKF01*ampG*For and KKF04*ampG*Rev (Table 3). Similarly, KKF05*ampP*For and KKF08*ampP*Rev were used to PCR-amplify a 2779-bp *ampP *fragment. The *ampP *and *ampG *PCR products were cloned into pCRII-TOPO according to the manufacturer's instruction (Invitrogen, CA), generating pKKF04 and pKKF03, respectively. A Gm cassette carrying the *aacCI *gene was retrieved from pUCGm [38]. The cassette was inserted into the unique *Hinc*II and *Asc*I restriction sites of *ampP *and *ampG*, respectively, creating pKKF145 and pKKF149 (Figure 2). These insertions created a polar mutation in the 5'-ends of *ampP *and *ampG *ORFs in pKKF04 and pKKF03, respectively. Subsequently, the *ampP*::*aacCI *and *ampG*::*aacCI *from pKKF145 and pKKF149, respectively, were sub-cloned into the *Sma*I site of pEX100T [39], a mobilizable suicide plasmid. These plasmids were conjugated into *P. aeruginosa *PAO1, with a helper strain harboring pRK2013 [40]. The merodiploids, resulting from homologous recombination, were selected with PIA containing Gm. These Gm^R ^colonies were then screened for Gm resistance and Cb sensitivity by replica plating. The insertions were confirmed by PCR and restriction analysis of the PCR product (data not shown). The PAO1 isogenic strains with defective *ampP *and *ampG *are henceforth referred to as PAO*ampP *and PAO*ampG*, respectively.

### Construction of *ampP *and *ampG *complementing plasmids

Plasmids containing *ampP *and *ampG*, pKKF73 and pKKF69, respectively, were generated by inserting the *Eco*RI fragment with *ampP *and *ampG *from pKKF004 and pKKF003 into a broad-host range, low copy number vector, pME6030 [41]. These were later conjugated into PAO*ampP *and PAO*ampG *for complementation analysis.

### Promoter-*lacZ *fusion constructions

The putative promoter regions of *ampG *and *ampP *were subcloned from pKKF003 and pKKF004 into pGEMEX-1, respectively, generating pKKF091 (P*_ampFG_-lacZ*) and pKKF087 (P*_ampOP_-lacZ*) (Table 3). This suicide vector contained the integration-proficient *attP *site, which recombines into the chromosomal *attB *site to generate a single-copy reporter fusion [42]. The resulting clones were mobilized into PAO1 and PAO*ampR *(Table 3). The presence of the chromosomal insertions was confirmed by PCR and restriction analysis of the product.

### Topological analysis of AmpP and AmpG

The topology of AmpP and AmpG were investigated using two markers, *phoA *and *lacZ*, that function in the periplasm and cytoplasm, respectively. The entire *ampP *gene was PCR amplified using primers KKF13*ampP2*For and KKF14*ampP2*Rev and cloned into pTrcphoA [43]. The entire *ampG *gene was PCR amplified using primers KKF09*ampG2*For and KKF10*ampG2*Rev and cloned into a pTrcphoA plasmid which had been modified by insertion of a broad host range stabilization fragment from pMF54 (Table 3). Both *ampG *and *ampP *genes were cloned into pTrclacZ [43]. The erase-a-base system (Promega, WI) was used to generate deletions of the genes from the 3'-ends. The resulting clones were then sequenced to determine the fusion junctions. The *phoA *and *lacZ *activities were determined as previously described [44].

### β-lactamase and β-galactosidase assays

β-lactamase and β-galactosidase activities were assayed as previously described [9,10].

### Determination of minimal inhibitory concentrations (MICs)

MICs were determined using E-test strips (Biomerieux, Marcy l'Etoile, France) according to the manufacturer protocols.

### Reverse transcription PCR

For the reverse transcription PCR, RNA was isolated from PAO1 using the RNAeasy mini kit (Qiagen, Valencia, CA) according to the manufacturer protocol. DNA was removed by two sequential 1 hour treatments at 37°C with RQ DNaseI (Promega Corporation, Madison, WI) followed by heat inactivation at 65°C for 10 minutes. Synthesis of cDNA was performed with Superscript III reverse transcriptase (RT) (Invitrogen, Carlsbad, CA) using a (NS)_5 _random primer and 5 μg RNA according to the manufacturer protocol. A control reaction containing all components except for Superscript III RT was performed in parallel. After cDNA synthesis, RNA was removed by treatment with 0.2 N NaOH for 30 minutes at 65°C. The reactions were neutralized by addition of 0.2 N HCl and cDNA was purified using the QIAquick PCR purification kit (Qiagen, Valencia, CA) according to the manufacturer protocol.

PCR reactions to amplify the *ampF-ampG *intergenic region were performed using primers PA4392_3junctionRTF and PA4392_3junctionRTR (Table 3) using GoTaq Flexi (Promega Corporation, Madison, WI). PCR reactions to amplify the *ampO-ampP *overlapping region were similarly performed with the exception that primers PA4218_9junctionRTF and PA4218_9junctionRTR (Table 3) were used. PCR products were analyzed by electrophoresis on a 10% polyacrylamide/1× TBE gel followed by staining with SybrSafe (Invitrogen, Carlsbad, CA).

## Authors' contributions

KFK identified the *P. aeruginosa ampG *orthologs, *PA4218(ampP) *and *PA4393(ampG)*, constructed the *ampG *and *ampP *insertional mutants, as well as the *lacZ *transcriptional fusion strains, performed the β-lactamase and β-galactosidase assays and prepared the first draft of the manuscript. AA constructed and assayed the LacZ and PhoA fusions. LS performed the reverse transcription PCR analysis, determined MICs and assisted with data analysis, figure preparation and wrote the submitted draft of the manuscript. KM conceived of the study, participated in its design and execution and helped in manuscript preparation. All authors read and approved the final manuscript.
